# P-1275. Effects of Clinically Relevant Antibacterial Agents on Type II Topoisomerases from Pseudomonas aeruginosa

**DOI:** 10.1093/ofid/ofaf695.1465

**Published:** 2026-01-11

**Authors:** Lauren Parr, Neil Osheroff

**Affiliations:** Vanderbilt University, Nashvilel, TN; Vanderbilt University School of Medicine, Nashville, TN

## Abstract

**Background:**

*Pseudomonas aeruginosa* is a gram-negative nosocomial pathogen with a growing number of multidrug-resistant infections. Treatment options, such as fluoroquinolones, are increasingly limited due to resistance. Fluoroquinolone resistance is driven primarily by single amino acid mutations in its target enzymes, gyrase and topoisomerase IV. These enzymes regulate DNA topology by introducing transient double-stranded breaks and passing another DNA segment through these breaks. Fluroquinolones kill cells by stabilizing the covalent enzyme-cleaved DNA complex, converting transient double-stranded breaks into breaks that can no longer be ligated by gyrase/topoisomerase IV. Gepotidacin, a triazaacenaphthylene, and zoliflodacin, a spiropyrimidinetrione, offer alternative treatments by targeting type II topoisomerases via distinct mechanisms, avoiding fluoroquinolone resistance mutations.

**Methods:**

The effects of fluoroquinolones, gepotidacin, and zoliflodacin on *P. aeruginosa* topoisomerase IV were assessed via DNA cleavage and catalytic activity inhibition.

**Results:**

Two fluoroquinolones, ciprofloxacin and moxifloxacin, displayed similar DNA cleavage activity (CC_50_ ∼3.0 µM, 30% DNA cleavage) but moxifloxacin was more potent in decatenation assays (IC_50_ ∼20 µM vs 29 µM). Gepotidacin displayed potent activity, inducing single- and double-stranded DNA cleavage (CC_50_ ∼0.01 µM and 0.02 µM, respectively) and catalytic inhibition (IC_50_ ∼15 µM). While CC_50_ values were similar, single-stranded DNA breaks exceeded 30%, while double-stranded breaks were ∼7%. Zoliflodacin displayed only moderate effects on both cleavage (CC_50_ ∼140 µM, 17% DNA cleavage) and catalytic inhibition (IC_50_ ∼480 µM). Three ParC mutants have been tested in DNA cleavage assays: two fluoroquinolone resistance mutants (parC^S87L^ and parC^E91K^) and a charge variant (parC^E91A^). For the fluoroquinolone mutants, only moxifloxacin and ciprofloxacin had significantly different CC_50_ values, and there were no significantly different CC_50_ values observed for parC^E91A^.

**Conclusion:**

Gepotidacin’s potent activity against both wild type and mutant enzymes, with lower CC_50_ and IC_50_ values than fluoroquinolones, supports its potential as a therapeutic option for drug-resistant *P. aeruginosa* infections.

**Disclosures:**

All Authors: No reported disclosures

